# Death of a Dog Caused by Ascarides

**Published:** 1880-10

**Authors:** 


DEATH OF A DOG CAUSED BY ASCARIDES.—H. Weiskoff (*Wochenschrift fur Thierheilkunde und Viehzucht*, No. 28, July, 1880), reports the following case: A young dog, seven weeks old, died suddenly without premonitory symptoms, and after it had shown good appetite and had frolicked about almost up to the time of its death. The owner of the animal suspected poisoning and a post-mortem was therefore requested of Weiskoff. On opening the abdomen a portion of the small intestines was found to be gangrenous, the alimentary canal here contained mucus, but no remnants of food. The jejunum containing the gangrenous patch was intensely inflamed for about the distance of one foot. At one point there was found a knot of coiled-up ascarides, which almost occluded the entire lumen of the gut. About seventy-five of these worms were found. The mucus membrane around this spot was greatly altered. It had a dark brownish red color, was considerably thickened and showed numerous furrows and canals of various shapes and sizes, the borders of these apparent passages were callous, ulcerative, and looked as though the intestine had been gnawed. The muscular coat of the intestine appeared to be involved in this morbid process at some places. The canals were occupied by the worms, which were covered over with slime and surrounded by ova. The stomach contained food in abundance, but no worms. There was some peritonitis; all the other organs of the animal were found to be normal.

